# Dietary Strategies in the Prevention and Treatment of Hypertension in Children and Adolescents: A Narrative Review

**DOI:** 10.3390/nu16162786

**Published:** 2024-08-21

**Authors:** Agnieszka Kozioł-Kozakowska, Małgorzata Wójcik, Vesna Herceg-Čavrak, Sara Cobal, Dragan Radovanovic, Julio Alvarez-Pitti, Isa Hartgring, Beata Piórecka, Rosita Gabbianelli, Dorota Drożdż

**Affiliations:** 1 Department of Pediatrics, Gastroenterology and Nutrition, Institute of Pediatrics, Faculty of Medicine, Jagiellonian University, Medical College, Wielicka 265 St., 30-663 Cracow, Poland; 2 Department of Pediatric and Adolescent Endocrinology, Chair of Pediatrics, Institute of Pediatrics, Faculty of Medicine, Jagiellonian University, Medical College, Wielicka 265 St., 30-663 Cracow, Poland; malgorzata.wojcik@uj.edu.pl; 3 University Children’s Hospital in Krakow, Wielicka 265 St., 30-663 Cracow, Poland; dorota.drozdz@uj.edu.pl; 4 Faculty of Health Sciences, Libertas International University, 10 000 Zagreb, Croatia; vherceg@gmail.com; 5 Croatian Medical Association, 10 000 Zagreb, Croatia; cobal.epode@hlz.hr; 6 Department of Medical Sciences, Faculty of Sport and Physical Education, University of Nis, 18000 Nis, Serbia; dragan.radovanovic@fsfv.ni.ac.rs; 7 Pediatric Department, Consorcio Hospital General, University of Valencia, 46014 Valencia, Spain; japnago@gmail.com; 8 CIBER Fisiopatología Obesidad y Nutrición (CIBEROBN), Instituto de Salud Carlos III, 28029 Madrid, Spain; 9 Innovation in Paediatrics and Technologies-iPEDITEC—Research Group, Fundación de Investigación, Consorcio Hospital General, University of Valencia, 46014 Valencia, Spain; isahartgring@gmail.com; 10 Department of Nutrition and Drug Research, Institute of Public Health, Faculty of Health Sciences, Jagiellonian University Medical College, Skawińska 8 St., 31-066 Cracow, Poland; beata.piorecka@uj.edu.pl; 11 Unit of Molecular Biology and Nutrigenomics, School of Pharmacy, University of Camerino, 62032 Camerino, MC, Italy; rosita.gabbianelli@unicam.it; 12 Department of Pediatric Nephrology and Hypertension, Chair of Pediatrics, Institute of Pediatrics, Jagiellonian University Medical College, Wielicka 265 St., 30-663 Crakow, Poland

**Keywords:** hypertension, blood pressure, obesity, children diet, DASH diet

## Abstract

This study aims to gather information on effective dietary strategies for the prevention and treatment of hypertension (HTN) in children and adolescents. It discusses specific nutritional models such as the Diet Approaches to Stop Hypertension (DASH diet), traditional Asian diets, plant-based diets, the Southern European traditional Atlantic diet, and the Mediterranean diet, highlighting the benefits of these approaches. The manuscript also addresses dehydration resulting from insufficient fluid intake among children, as well as the consumption of inappropriate beverages, like soft drinks and energy drinks, which contributes to the development of HTN. Additionally, it examines the role of oxidative stress in the pathomechanism of HTN in children, particularly in relation to the antioxidant potential of food components such as selenium, magnesium, and selected vitamins. The relationship between sodium and potassium intake from food and the development of HTN in children is also explored. Finally, this study discusses public health strategies for the prevention of HTN in children. A comprehensive search was performed across multiple databases, such as PubMed/MEDLINE, the Cochrane Library, Science Direct, and EBSCO. This search focused on locating English-language meta-analyses, systematic reviews, randomized clinical trials, and observational studies from around the globe.

## 1. Introduction

Hypertension (HTN), a condition traditionally associated with adults, is increasingly being diagnosed in children and adolescents. The rise in pediatric hypertension is a concerning public health issue, closely linked to the escalating rates of childhood obesity and lifestyle changes [1]. Hypertension in childhood can lead to the early onset of cardiovascular diseases and other serious health complications later in life [1,2]. Early detection and management are crucial in preventing these long-term consequences. Diet plays a pivotal role in the prevention and treatment of hypertension. The focus on dietary interventions is driven by the understanding that certain dietary patterns can significantly influence blood pressure, either directly or indirectly, through their effects on obesity. Dietary strategies, especially those involving dietary modifications, are among the most effective and noninvasive approaches to blood pressure control and hypertension prevention. Among the most well-known Diet Approaches to Stop Hypertension (DASH diets), there are at least a few other eating patterns that can have a potential effect on blood pressure [3]. Can these nutritional approaches be recommended in the fight against hypertension in children? What other elements of the diet, such as the type of fluids, the amount of salt consumed, or perhaps antioxidants in food, are important in hypertension? The purpose of this manuscript is to discuss various models of nutrition with better- or lesser-known impacts on the development of arterial hypertension and to critically refer to them, as well as to discuss the mechanisms involved in nutrients and their potential effects on HTN. This manuscript also addresses the issue of adequate water intake in relation to the development of HTN, as well as the effects of other beverages, such as sweetened soft drinks and energy drinks, on the development of HTN in children and adolescents. We review the current evidence on specific nutrients, dietary patterns, and public health strategies that can help manage blood pressure in the pediatric population. Understanding the relationship between diet and hypertension is essential for developing comprehensive prevention and treatment strategies. By addressing the dietary factors contributing to high blood pressure (BP), we can create more effective and sustainable health interventions for children at risk of or suffering from hypertension. This approach not only improves their immediate health but also reduces their risk of cardiovascular diseases and other related complications in adulthood.

## 2. Material and Methods

A search was conducted across several databases, including PubMed/MEDLINE, the Cochrane Library, Science Direct, and EBSCO. The search targeted English-language meta-analyses, systematic reviews, randomized clinical trials, and observational studies worldwide. Additionally, the websites of scientific organizations like the World Health Organization (WHO) and the European Food Safety Authority (EFSA) were examined. The articles selected were mutually agreed upon by all authors, with a particular focus on meta-analyses, systematic reviews, and content of interest to the pediatric medical community.

## 3. DASH Diet: Is It Still Relevant?

Healthy nutritional models, such as the DASH diet, are increasingly growing. Developed in the 1990s to treat high blood pressure in adults without medication, the DASH diet emphasizes low sodium and saturated fat in foods and beverages. It encourage consuming more whole grains, fruits, vegetables, nuts, seeds, legumes, and low-fat dairy while reducing processed meats, salty foods, and sweetened drinks. The DASH diet also emphasizes the consumption of minimally processed and fresh food [3]. Studies have shown that the DASH diet leads to a higher intake of minerals such as potassium (K), magnesium (Mg), and calcium (Ca), as well as fibers and other bioactive components contained in vegetables and fruits [4].

**Figure 1:**
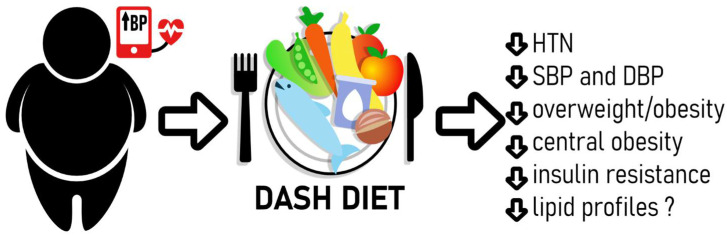
The benefits of implementing the DASH diet for children and adolescents with excess body weight and elevated blood pressure.

The results of the meta-analysis by Cheng et al. from 2015 concluded that the DASH diet had a positive clinical effect on BP in children and adolescents and caused a greater decrease in systolic blood pressure (SBP) than in diastolic blood pressure (DBP) [5]. Current clinical practice guidelines advocate for a DASH diet for BP management in youth [6]. The recent randomized controlled trial conducted by Couch et al. evaluated the effects of a 6-month DASH diet-focused behavioral nutrition intervention on BP and endothelial function in adolescents with elevated BP. The results demonstrated that the DASH group showed significant improvements in SBP, endothelial function, and diet quality immediately postintervention and at the 18-month follow-up compared to those receiving routine care [7]. In a Dutch cohort study of 1068 children aged 5 to 7, researchers examined the link between adherence to the DASH diet, genetic risk factors, and BP. The study found that every 10-unit increase in DASH score was associated with a 0.7 mm Hg decrease in SBP. Conversely, each standard deviation increase in genetic score was linked to a 0.5 mm Hg increase in DBP. A significant gene–diet interaction shows that low DASH diet adherence combined with high genetic risk can worsen DBP in children. This indicates that both diet and genetic factors influence BP in the pediatric population [8]. The DASH diet may prevent overweight in periadolescents. In a population of 1570 schoolchildren aged 12.4 from Ontario (Canada), the DASH index was associated with decreased body composition measures in both genders. Specifically, the DASH index was negatively associated with BMI, WHtR (waist-to-height ratio), WHR (waist-to-hip ratio), WG (waist girth), and HG (hip girth). A higher DASH index was also associated with lower odds of being overweight in girls and boys [9]. However, a recent study by Bricarello et al. no found significant association between adherence to the DASH diet and overweight/obesity among Brazilian adolescents [10]. The systematic review developed by Bricarello et al. (2018) concluded that the DASH diet may benefit the alterations of BP and excess body weight in adolescence. The review showed that the DASH diet leads to reduced DBP and lower BMI increases over 10 years. However only one randomized controlled trial (RCT) demonstrated its effectiveness in lowering SBP; another RCT noted a decrease in the prevalence of arterial hypertension (AH) [4]. In a recent review about delaying the onset of or reducing adverse health outcomes related to high blood pressure in children and adolescents, the DASH-type diet achieved statistically significant reductions in SBP by 2.2 mmHg and in DBP by 2.8 mmHg. These results were noted in a completers-only analysis of one fair-quality RCT [11]. The study by Louca et al. on a group of 2424 women demonstrated that BMI plays a key role in mediating the relationship between the DASH diet and hypertension, explaining 39.1% of the variance in the occurrence of this condition, and suggested that weight reduction may contribute to lowering BP [12]. Figure 1 illustrates the benefits of implementing the DASH diet for children and adolescents with excess body weight and elevated blood pressure.

Findings from studies that investigated the effect of the DASH diet on lipid profiles present contradictory results. Asghari et al. explored how closely following a DASH-style diet is linked to the occurrence of metabolic syndrome (MetS) in 425 children and adolescents. There was a statistically significant link between the DASH diet and lower levels of high fasting plasma glucose, hypertension (HTN), and reduced waist circumference (WC). However, they found no significant connection between triglyceride and high-density lipoprotein cholesterol (LDL) levels [13]. A clinical trial by Saneei et al. reported that compliance with the DASH diet for 6 weeks could decrease the prevalence of HTN in adolescents with MetS [14]. Saneei et al. also examined the effects of the DASH diet on markers of systemic inflammation in adolescents with MetS. Consumption of the DASH eating pattern may reduce circulating levels of hs-CRP among adolescents; however, other inflammatory markers were not affected by the DASH diet [15]. Chiu et al., in another RTC among adolescents, found that adhering to the DASH diet significantly reduced LDL, high-density lipoprotein cholesterol (HDL), and apolipoprotein A-I (apo A-I). The study was conducted in free-living healthy individuals who consumed in random order a control diet, a standard DASH diet, and a higher-fat, lower-carbohydrate modification of the DASH diet (HF-DASH diet) for 3 wk each. The HF-DASH diet lowered BP to the same extent as the DASH diet but also reduced plasma triglyceride and VLDL concentrations without significantly increasing LDL cholesterol [16]. A recent cohort study conducted in Mexico among children and young adults reported that a higher DASH score was associated with a decrease in insulin resistance but not other cardiometabolic markers, although combined cardiometabolic risk was not assessed [17]. The greatest compliance to the DASH diet was linked to reduced odds of hyperglycemia, hypertriglyceridemia, low HDL-cholesterolemia, and insulin resistance. Considering the inverse relationship observed between the DASH diet and the metabolically unhealthy obesity (MUO) phenotype in the survey conducted by Heidari et al., it is clinically valuable to advise adolescents to increase the consumption of health-related components of the DASH diet [18]. There are limited studies on the long-term effectiveness of the DASH diet. The study by Winpenny et al. examined dietary patterns from 2957 participants aged 13 to 30, collected through the National Diet and Nutrition Study (2008–2016), to explore changes in diet quality during the progression from late adolescence to early adulthood. This study found no significant alterations in diet quality (DASH index) among males; however, improvements were observed in females within specific age groups [19]. The study by Krijger et al. explored how diet quality at ages 5–6 impacts cardiovascular health by ages 11–12 in a diverse cohort from the ABCD study, assessing diet through the DASH score and Child Diet Quality Score (CDQS). At follow-up (N = 869), they examined BMI, WC, BP, lipid levels, fasting glucose, and carotid intima-media thickness. Findings indicated that higher diet quality was associated with better outcomes in BMI, WC, both SBP and DBP, and triglycerides. There were no significant associations between diet quality and LDL-C, HDL-C, total cholesterol, fasting glucose, or carotid thickness, suggesting that good early childhood diet quality can positively affect some cardiovascular risk factors by preadolescence [20]. Participants in the ALSPAC cohort study, assessed at ages 7, 10, 13, 17, and 24, were evaluated for adherence to a DASH-style diet using the DASH Dietary Score (DDS). While no significant associations were found between DDS at ages 7, 10, and 13 and blood lipids or blood pressure at age 17, a higher DDS early in life was linked to better glucose metabolism and lower BMI at ages 17 and 24. Additionally, better adherence to the DASH diet at age 7 was associated with lower DBP and higher HDL cholesterol at age 24. These findings suggest that adhering to a DASH-style diet during childhood could improve cardiometabolic health into adulthood [21]. As obesity and metabolic syndrome rates continue to increase in children and adolescents, emphasizing healthy dietary habits is a crucial intervention. The DASH diet remains relevant as an effective dietary model for managing hypertension and improving overall cardiovascular health.

## 4. Dietary Patterns in the Prevention and Treatment of Hypertension

**Figure 2:**
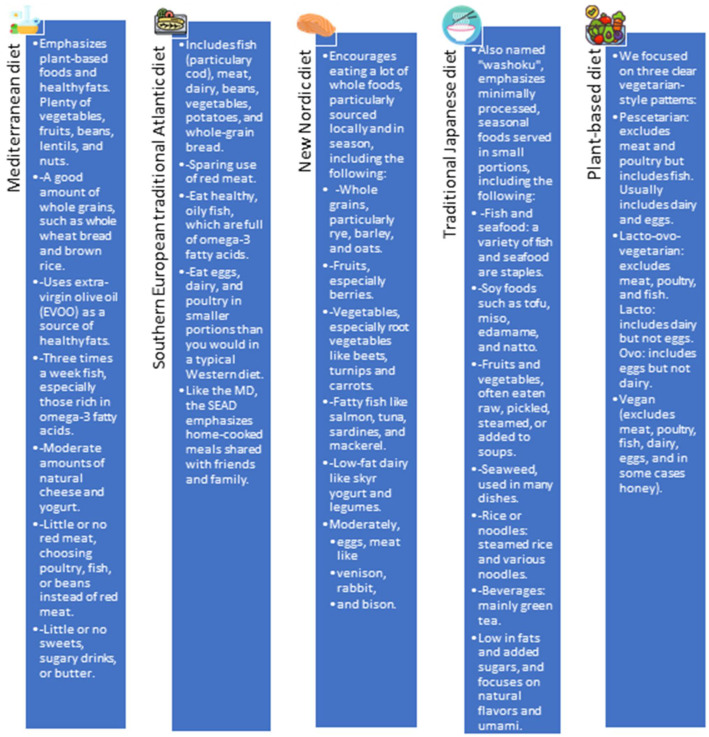
Main characteristics of described diet patterns.

DASH is undoubtedly one of those with the most evidence regarding its efficacy in the prevention and treatment of HTN in adults [3] and in children and adolescents [7]. However, there are other dietary patterns that impact BP values, although the information availability is scarce and mostly based on low-quality studies. Characteristics of each pattern are summarized in Figure 2.

Mediterranean diet: Previous studies in Caucasian children have shown that the lower the adherence to the Mediterranean diet (MD) pattern, the higher the degree of obesity [22], and that the introduction at an early age of this diet pattern can prevent the development of overweight and obesity in childhood [23]. Nevertheless, few studies have reported on the effects of MD adherence on blood pressure (BP). Sub-studies of the PREDIMED trial indicate that an MD supplemented with olive oil or nuts leads to reductions in SBP by 5.8–7.3 mmHg and DBP by 3.3–3.4 mmHg in adults compared to a low-fat control diet [24]. In a study carried out in Spain by López-Gil et al. on a sample of 698 adolescents (12–17 years), body composition and BP were measured according to the recommendations of the EHS [25]. The degree of adherence to the MD was evaluated using the KIDMED questionnaire, and the habit of napping was also assessed. Subjects with low or moderate adherence to MD were more likely to have high-normal BP or HTN than those with high adherence (*p* = 0.007). However, no statistically significant differences in systolic and DBP values were found between the two groups. Also, there was no significant difference in BP between those who did and those who did not take a nap (*p* = 0.071). When considering both behaviors, a higher frequency of high-normal BP or HTN was observed in adolescents who did not follow either behavior than in those who did (*p* = 0.012). Results showed lower SBP (β = −2.60 mmHg; 95% CI: −5.18–0.02) and DBP (β = −1.65 mmHg; 95% CI: −4.00–0.71) in subjects with high MD adherence and frequent naps compared with those with low adherence to MD and no naps, but only the reduction on SBP was statistically significant. As part of the Healthy Lifestyle in Europe by Nutrition in Adolescence (HELENA) multicenter and cross-sectional study, a sample of 605 adolescents (51.6% female) had their body composition, BP (following EHS guidelines), and adherence to the MD evaluated among other variables [26]. Multilinear regression models showed that MD adherence protected against increasing BMI, waist circumference, and metabolic syndrome in both sexes. They found only in males an association of MD with mean arterial pressure (*p* ≤ 0.05) and DBP (*p* < 0.05), while in females they just observed an association with HOMA and SBP. This information was analyzed using genetic risk scores (GRSs), which combined several single-nucleotide polymorphisms (SNPs) by summing the number of risk alleles, and it was found that male adolescents with high adherence to the MD and fewer genetic risk alleles had lower DBP. Following this approach, in a recent study involving 548 adolescents from the HELENA cohort (53% female), researchers examined the impact of an MD score and a genetic risk score for hypertension (HTN-GRS) based on 16 single-nucleotide polymorphisms (SNPs) associated with HTN [27]. The results showed that adherence to the MD had a protective effect on both systolic and DBP z-scores. However, this protective effect decreased as the number of risk alleles in the HTN-GRS increased. Additionally, the effect was greater in females than in males. Based on this knowledge, it can be concluded that there is a relationship between a high adherence to a MD in children and adolescents and lower BP values, but there are different responses depending on the genetic load of each individual.

Southern European Traditional Atlantic Diet (SEAD)

The effect on cardiometabolic health in adults and children was analyzed in the GALIAT Atlantic Diet Study [28]. Families were divided into two groups as part of the GALIAT trial. One group followed the SEAD protocol. They were also taught about the diet and given cooking classes to increase their adherence. The control group followed their usual diet and lifestyle. The study was completed by 213 families (92.4%). Adults in the intervention group lost weight as opposed to controls, who gained; also, their total cholesterol and low-density lipoprotein cholesterol levels were significantly reduced in comparison with controls. No differences in other lipids, glucose metabolism profile, or BP were observed [29]. No changes were observed among the participating children.

New Nordic diet (NND)

A recent meta-analysis of three controlled trials involving 420 adults found that consuming a healthy Nordic diet significantly reduced SBP by 4.47 mmHg and DBP by 2.32 mmHg compared to regular diets, with moderate certainty in the results [30]. In the prospective randomized controlled trial STRIP (Special Turku Coronary Risk Factor Intervention Project; *n* = 877 children), biannual dietary counseling was provided based on the Nordic Nutrition Recommendations. After 20 years of follow-up, achieving a higher number of dietary targets was linked to lower SBP (mean [SE] SBP: 107.3 [0.3], 107.6 [0.3], 106.8 [0.3], and 106.7 [0.5] mmHg in participants meeting 0, 1, 2, and 3 to 4 targets, respectively; *p* = 0.03) and lower DBP (mean [SE] DBP: 60.4 [0.2], 60.5 [0.2], 59.9 [0.2], and 59.9 [0.3] mmHg; *p* = 0.02) in participants meeting 0, 1, 2, and 3 to 4 targets, respectively [31]. In Finland, Meinila J et al. tried to analyze in a cross-sectional retrospective study if the correlation between the Nordic diet and BP can be modified by the variable of birth weight, based on the idea that low birth weight is a risk factor for developing HTN. After a 10-year follow-up, a significant interaction between birth weight and ND was observed on SBP, especially in participants with a low birth weight (women < 2951 g, men < 3061 g). On the contrary, this was not the case for DBP and mean arterial pressure. Therefore, it seems that through a healthy diet (in this case ND), benefits on SBP can be measured, even more so in individuals with low birth weight [32].

Traditional Asian diets (Japanese, Korean Chinese)

In a 3-year longitudinal study made among Japanese adults, adherence to a traditional Japanese diet (TJD) was associated with favorable effects on BP [33]. In the 2012 Japan National Health and Nutrition Survey, following a TJD versus a Western or meat- and fat-based dietary pattern was found to have a lower prevalence of HTN in men [34]. However, this diet was associated with higher DBP in women, as well as higher waist circumference and BMI in men. Similar results were found in an epidemiological study based on a Korean traditional diet, suggesting a reduced risk of metabolic syndrome (odds ratio [OR]: 0.77; 95% CI: 0.60–0.99), obesity (OR: 0.72; 95% CI: 0.55–0.95), hypertension (OR: 0.74; 95% CI: 0.57–0.98), and hypertriglyceridemia (OR: 0.76; 95% CI: 0.59–0.99) [35]. Less evidence is found about the traditional Chinese diet.

Plant-based diets (PBDs)

Several cross-sectional studies have been published analyzing the impact of different PBD on body weight, macronutrient intake, and lipid profile. In general, PBD improves BW and lipid profile vs. an omnivorous diet, but by contrast, some macro- and micronutrient deficiencies (mainly 25-hydroxy vitamin D, serum iron, and vitamin B12) are observed in children following PBDs. This is the reason why the European Society of Pediatric Gastroenterology, Hepatology and Nutrition (ESPGHAN) does not recommend a vegan diet for the pediatric population but highlights that if a parent chooses to wean an infant onto a vegan diet, this should be performed under regular medical and expert dietetic supervision, and parents should receive and follow nutritional advice [36]. Nevertheless, two prospective randomized trials showed the efficacy of PBDs in lowering BP in children and adolescents, both conducted by Macknin et al. [37,38]. The most important was performed in 96 patients with obesity and hypercholesterolemia, in which the effects on cardiovascular risk factors of three different diet patterns (PBD, in which daily vitamins B12 and D were supplemented; MD; and American Heart Association (AHA)-advised healthy diet [39]) were assessed. The results showed similar, statistically significant (*p* < 0.05 to <0.001) improvements in all groups: myeloperoxidase, LDL-C and total cholesterol plasma levels, weight, and SBP and DBP were all reduced [38]. In those participants who followed the PBD, after 4 weeks of intervention, SBP decreased significantly by −6 mmHg (−l6, 0), *p* = 0.009, as did diastolic BP by −1 mmHg (−10, 0), *p* = 0.019.

As can be observed, although these dietary patterns come from different regions, with different cultures, the healthy dietary patterns had a similar composition. In general, they were characterized by the following food groups: fruits, vegetables, whole grain, fish, eggs, and nuts.

Unfortunately, there is insufficient evidence in children and adolescents to compare the efficacy of each of these dietary patterns in terms of their impact on BP values or other cardiovascular risk factors [40].

There are other types of diets that may have an impact on body composition and BP such as a low-carb diet, ketogenic diet, high-protein diet, or paleolithic diet. We decided not to include them in the review because they are not free of side effects, are not nutritionally balanced, and are not considered healthy eating patterns.

## 5. Role of Sodium and Potassium

One of the most important, well-documented dietary-associated factors causing the development of obesity and arterial hypertension is sodium intake [41]. However, it is not only daily sodium dose but also its ratio to potassium intake that matters. The relative potassium deficiency (in relation to the amount of consumed sodium) seems to be more important as the cause of hypertension than the absolute amount of sodium in the diet itself [42]. The main form of sodium intake is sodium chloride (NaCl), which is simply table salt. Other compounds added during the processing of food products, such as sodium monoglutamate, may also be a source of sodium. Generally, the leading source of sodium is industrially processed and ultraprocessed food, accounting for 71% of total sodium intake [43]. Many studies have shown that sodium intake in children is between 100% and 250% higher than the recommended dose. Moreover, as demonstrated in some studies, excessive dietary sodium intake prompts thirst, which might drive increased consumption of sugar-sweetened beverages [44]. In contrast, dietary potassium is obtained largely in minimally processed foods including fruit and vegetables, meat, and dairy products [45,46,47]. Although there are recommendations for adequate intake (AI) of sodium and potassium for children, it is important to remember that the data used to develop them do not come from experiments but are extrapolated from recommendations for adults. There are also minor discrepancies between the recommendations regarding AI provided by various organizations (e.g., WHO, US AGR Dep). Table 1 below presents sample recommendations by the European Food Safety Authority (EFSA) [46,47].

The role of sodium intake in regulating blood pressure is multifactorial. The fundamental element is its role as the main cation of the extracellular space, pivotal in determining fluid balance [48]. However, the regulatory role of sodium goes far beyond simple volumetric mechanisms. Several factors modulate salt handling such as activation of the sympathetic nervous system, hyperinsulinemia, hyperleptinemia, hyperaldosteronism, hypercalcemia, acid–base balance, vitamin D, and genetic background. Most of these systems ultimately work through the kidney, with the renin–aldosterone–angiotensin system playing a pivotal role in fluid and sodium homeostasis. Some recently published studies indicate the existence of many complex mechanisms leading to the development of salt-sensitive hypertension in obese individuals. According to the classical theory of salt-sensitive arterial hypertension development, a strict salt restriction should be of universal benefit [41]. Contrary to sodium, most of the body’s potassium is located in the cells. The role of potassium in the regulation of BP is also complex. Its transport across the membranes of the endothelial and vascular smooth muscle cells has important effects on their contractile state, which can, in turn, influence endothelial function, blood flow, and BP [41,49]. The concentration of potassium in cells of the collecting duct system of the kidney is important for the excretion of sodium. Maintenance of the transmembrane gradient is the key element for electrolytes and fluid homeostasis, a critical factor in blood pressure regulation [50].

A number of the excellent-quality systematic reviews of randomized controlled trials (RCTs) concluded that decreased sodium intake relative to usual or higher intake results in lowered blood pressure in adults with or without hypertension [51,52,53,54]. According to the meta-analysis performed by the WHO of nine controlled trials conducted in children with 14 comparisons testing systolic blood pressure, decreased sodium resulted in a decrease in resting systolic blood pressure of 0.84 mmHg (95% CI: 0.25, 1.43) [55].

## 6. Fluid Intake

Among the various beverages available, the choice between water and other liquids is particularly significant in the context of preventing HTN in children. Water is the most essential and beneficial beverage for maintaining optimal health and preventing HTN. Adequate water intake ensures proper hydration, which is vital for maintaining blood volume and preventing dehydration, a condition that can potentially raise BP. It is known that mild dehydration is associated with kidney dysfunction, which consequently leads to the development of hypertension [56]. Available data indicate that most children fall short of the recommended daily fluid intake; over 80% of children in many European countries consume less water than the EFSA guidelines recommend, and 54.5% are inadequately hydrated [57]. In contrast, consumption of other beverages like soft drinks (SSBs) and sport or energy drinks have negative impacts on HTN, yet their consumption is rising [58]. Recent meta-analyses have provided valuable insights into the relationship between sugar-sweetened beverages (SSBs) and the risk of hypertension and also obesity in children. High consumption of SSBs has been shown to elevate SBP and increase the risk of HTN among children [59,60,61,62]. One comprehensive meta-analysis found that high consumption of SSBs is significantly associated with an increased risk of both HTN and obesity in children and adolescents. The study reviewed data from various research articles and concluded that SSB intake negatively affects weight and increases the risk of metabolic diseases such as HTN. SSB consumption was associated with a 1.67 mmHg increase in SBP in children and adolescents but not with a significant rise in DBP. High SSB consumers were, moreover, 1.36 times more likely to develop HTN compared with low SSB consumers. The primary mechanism is linked to decreased satiety and incomplete compensatory reduction in energy intake after consuming liquid calories, which contributes to weight gain and subsequent metabolic complications [63]. Gallagher et al. evaluated a sample of 2665 Greek schoolchildren, assessing body composition, anthropometric parameters, dietary intake, and serum cortisol data, and observed a positive association between high SSB consumption (>2 servings per day) and visceral adipose tissue, which is correlated with HTN [64]. In Sakaki’s study, 9.043 participants (9–16 years) were involved over an average follow-up period of 17 years. Among them, there were 618 cases of HTN. The study found that consumption of SSBs, but not fruit juice, was linked to HTN [65]. There is consistent evidence indicating that the type of sugars consumed can affect BP and vascular function, with noticeable effects often seen shortly after intake. Pure fruit juices, such as orange, grapefruit, and grape juice, as well as whole fruits, generally elicit positive responses, including lower blood pressure and improvements in flow-mediated dilatation and microvascular endothelial reactivity. These benefits are not typically observed with SSBs and honey. Studies have also shown enhanced endothelial function with whole fruits, although this did not lead to additional reductions in blood pressure [66]. Replacing SSBs with water not only helps in reducing overall calorie intake but also promotes better hydration, which is crucial for maintaining healthy blood pressure levels. This shift in fluid consumption can be a key intervention in preventing hypertension in children [67].

Among many fluids, energy drinks and sports drinks are becoming more and more popular but unfortunately may have an impact on HTN in children and adolescents. Sports drinks are flavored liquids that often contain additional vitamins and minerals, electrolytes, carbs, and minerals. They might help young athletes who engage in high-intensity or endurance sports regularly as well as those who exercise for longer than an hour or in extremely hot or humid weather [68]. However, since sugar is included in their list of ingredients, sports drinks should not be a substitute for water. According to scientific studies, 30 and 50% of adolescents reported using energy drinks [69,70] despite the American Academy of Pediatrics advising teenagers between the ages of 12 and 18 not to drink energy drinks and to limit their daily caffeine intake to 100 mg or an average cup. In children and adolescents, excessive consumption of energy drinks may result in HTN, tachycardia headaches, palpitations, anxiety, and sleeplessness [71]. In healthy youths, acute energy drink use is linked to significantly increased systolic and diastolic blood pressure, along with a possible decrease in heart rate [72]. The use of energy drinks containing stimulants such as caffeine, guarana, and others might also be linked to anxiety, insomnia, gastrointestinal issues, and dehydration. Energy drink usage may be particularly harmful in situations where they are combined with other risk factors (such as drug use or physical activity) or when there are underlying medical issues [73]. A recent study reported that in healthy children and adolescents, consuming a single bodyweight-adjusted energy drink in the morning is associated with considerably increased 24 h blood pressure, both diastolic and systolic [74]. Along these lines, energy drink use throughout the years may increase the risk of cardiovascular disease in youths. An additional influence on HTN in adolescents may be the practice of combining energy drinks with alcohol, which results from two trends that have occurred in the last twenty years: the increase in the consumption of energy drinks and the increase in alcohol intake. Energy drinks mixed with alcohol lead to higher overall alcohol intake, which is usually explained by the caffeine’s stimulant properties offsetting the alcohol’s depressing properties. Energy drinks’ high caffeine concentration makes youths feel the need to consume more alcohol, which frequently results in alcohol drunkenness. Energy drink consumption combined with alcohol use is a serious problem, particularly in adolescence, as it can have an impact on risk-taking behavior and overall health [75,76]. Considering the potential long-term detrimental impacts on youth health, the first step should be to determine the upper limits of acceptable caffeine use [77]. Therefore, it is necessary to raise public awareness about the possible dangers of abusing energy drinks and other sugar-sweetened beverages in an effort to encourage young people to make wise choices about how much they will consume.

## 7. Antioxidants from Food and Their Role

Oxidative stress and inflammation have been associated with endothelial damage and vascular stiffness, suggesting that coping with stress could represent a strategy to prevent HTN and cardiovascular diseases. Endothelial cells play a very important role in regulating vascular homeostasis. Endothelial cells act as a physical barrier between blood and tissues and produce substances and mediators which exhibit antithrombotic and anti-inflammatory properties. The main mediator of vasodilation is nitric oxide (NO) released from endothelial cells, which, through various mechanisms, affects vascular smooth muscle cells and causes their relaxation. NO also plays other important protective roles for the endothelium and vessels—it inhibits platelet aggregation and adhesion and is involved in smooth muscle cell proliferation, leucocyte adhesion, vascular permeability, and inflammatory mechanisms and has antiatherogenic properties. Reactive oxygen species (ROS) significantly eliminate NO production. Reduced production and bioavailability of NO are the main mechanism of the influence of endothelial dysfunction on vasoconstriction, which, via increasing peripheral resistance, influences the development of arterial hypertension. Oxidative stress and inflammation are conditions that damage the endothelium and shift endothelial function from vasoprotective to vasoconstrictive [78]. Oxidative stress occurs when an impairment between the production of free radicals and the level of antioxidants arises; free radicals are produced physiologically following cell metabolism; oxygen is reduced to water during oxidative phosphorylation. However, part of the oxygen (about 2%) can be partially reduced and released as superoxide anion and/or hydroxyl radical. The endogenous redox system, constituted by Mn-superoxide dismutase (SOD) in mitochondria and Cu/Zn-SOD, catalase (CAT), and glutathione peroxidase (GPx) in the cytosol, works to reduce these high reactive oxygen species into water. The contribution of exogenous antioxidants in food represents a key opportunity to improve the redox system of the organism. Several foods rich in polyphenols are able to activate the transcription factor Nrf2/ARE pathway, a modulator of antioxidant and detoxicant genes, and inhibit the KF-kB transition factor pathway activator of pro-inflammatory cytokines [79]. 

The daily use of vegetables and fruit during meals, in a correct amount, can be guaranteed to have the required quantity and quality of antioxidants useful in coping with physiological ROS production. Sulforaphane (SNF) from broccoli is an isothiocyanate able to inhibit COX-2 and promote Nrf2/ARE activation; broccoli and, particularly, broccoli sprouts contain SNF. Noteworthy is that SNF can increase NO production, which regulates systemic BP [80]. A recent study on the NHANES cohort (n = 21,526) identified a negative linear association between dietary antioxidant index and HTN [81]. Thus, hydrosoluble vitamin C (in kiwi, citrus fruits, strawberries, bell peppers, broccoli, and Brussels sprouts) and liposoluble vitamin E (in nuts, seeds, vegetable oils, spinach, broccoli) exert a potent antioxidant capacity in the hydrophilic–hydrophobic regions contributing to coping with stress. Food like carrots, sweet potatoes, pumpkins, spinach, kale, and butternut squash contain beta-carotene, while tomatoes, watermelon, pink grapefruit, red peppers, and papaya contain lycopene. 

Selenium, manganese, and zinc are cofactors of endogenous antioxidant enzymes, the GPx and the mitochondrial and cytosolic SOD, respectively [80]. A recent meta-analysis of treatments with vitamins E, C, B_2_, and folic acid evaluated whether their role is essential in HTN, and only vitamin E showed usefulness in reducing SBP [82]. However, it has to be highlighted that vitamin E, as well as the other liposoluble vitamins (A, D, and K), exerts a toxic effect if used as a supplement in a condition outside of hypovitaminosis; for this reason, intake of vitamin E should be derived from dietary products, and supplementation should occur only under medical control. Furthermore, it is necessary to consider that for antioxidants to work, they must be present at low concentrations, because an excess of antioxidants has been correlated with pro-oxidant impact.

Due to its antioxidant and anti-inflammatory properties, spirulina, a blue-green alga, was able to decrease blood pressure in humans with type 2 diabetes as well as improve plasma lipid profile; spirulina activates antioxidant-scavenging enzymes (SOD, CAT, GPx) and reduces the pro-inflammatory cytokines [83]. The flavonoid quercetin, contained in several fruits (i.e., apples, berries, cherries, citrus fruits, red grapes), vegetables (i.e., red leaf lettuce, onions, asparagus, tomatoes, broccoli, peas, pepper), tea, and wine, activates Nrf2, inhibits NF-kB, and acts as an antioxidant, increasing the glutathione level and membrane sulfhydryl groups [84]. Recently, Popiolek-Kalisz and Fornal in a meta-analysis indicated that appropriate supplementation with quercetin decreases SBP in normotensive subjects and decreases DBP in the (pre)hypertensive group [85]. Clinical studies have shown the beneficial effect of a healthy diet, rich in vegetables and fruits, on reducing the risk of cardiovascular diseases in adults. The meta-analysis of 14 studies reporting data from 1930 participants showed a beneficial effect of the Mediterranean diet on endothelial function [86]. In a clinical study after a 4-week run-in period during which fruit and vegetable intake was limited to one portion per day, participants were randomized to consume either one, three, or six portions daily for the next 8 weeks. For each one-portion increase in reported fruit and vegetable consumption, there was a 6.2% improvement in forearm blood flow responses to intra-arterial administration of the endothelium-dependent vasodilator acetylcholine [87]. However, it should be considered that an excess in the consumption of fruit should be avoided due to the negative impact of the fructose contained in fruit and that the consumption of vegetables and fruit has to follow the indication of the Mediterranean diet that includes three portions/day of vegetable and two portions/day of fruits. However, the European Society of Hypertension’s panel of experts suggested the use of nutraceuticals (beetroot juice, tea, and coffee as antioxidant-rich beverages; magnesium; potassium; vitamin C and resveratrol) in patients with high-normal BP, but their consumption should be derived from food, and when necessary, their supplementation has to be only in a deficit status of antioxidants (i.e., subjects with hypovitaminosis that do not eat vegetables or fruit) [88].

In conclusion, a diet rich in bioactive compounds with antioxidant and anti-inflammatory properties is recommended while avoiding the negative impact of antioxidant supplementation. The protective effects of a healthy diet are evident in the early stages of vascular damage, with important implications for the early prevention of cardiovascular diseases.

## 8. (In)appropriate Public Health Strategies—Effectiveness of Current Concepts

Evidence shows that childhood obesity positively correlates with cardiometabolic morbidity including HTN, stroke, and ischemic heart disease. The prevalence of HTN in children and adolescents with obesity is 5–30% and increases with higher BMI and age [89]. Obesity and hypertension in children, especially adolescents, are closely linked.

The development of obesity is a complex process, and the underlying mechanisms influencing the change from healthy weight to obesity and often elevated blood pressure are yet to be fully understood [90].

Thus, preserving healthy weight from an early age represents a key public health strategy [91,92]. Since obesity increases the risk of elevated blood pressure, it is necessary to implement prevention strategies for both conditions simultaneously. 

A prerequisite for reducing the risk of obesity and high blood pressure is the formation of healthy habits related to nutrition and physical activity (PA) from an early age [93]. Studies have identified several factors associated with the development of obesity, including poor dietary habits such as skipping breakfast, low intake of fruits and vegetables, consumption of foods and drinks high in sugar and sodium, and physical inactivity [94].

Developing an effective public health prevention strategy and intervention for hypertension is not a straightforward process, but it is of high priority and is a necessary approach for decreasing its prevalence among children and adolescents [95,96]. There has been a growing amount of public health research and interventions focusing on preventing lifestyle-related diseases, including hypertension, through family, school-based, and community-based interventions [97]. These studies vary in settings, single or multiple components (e.g., focus on diet, physical activity, and behavior change techniques), combinations of the aforementioned, and length of duration.

Family-based interventions

Parents play a crucial role in forming a child’s behaviors related to healthy lifestyle habits [95,97,98]. Research clearly shows that unhealthy lifestyle habits, unhealthy eating patterns, and reduced physical activity correlate with higher body weight in children and are linked with the development of HTN and cardiovascular disease in the long run [99,100].

In addition, research results on childhood obesity prevention with a home-based approach presented a BMI *Z*–score reduction of 36.99% (z = 36.99, *p* = 0.00) and a positive change in behavior related to healthy weight promotion among children [97]. Also, systematic reviews have reported on the valuable role of family-based interventions in obesity management of children with BP or HTN [101].

However, there is evidence that long-term adherence of parents and families to obesity prevention recommendations is difficult to attain and depends on various socioeconomic factors [95].

School—based interventions

Several studies highlight the effectiveness of school-based interventions focused on elementary and secondary school pupils for preventing childhood obesity [95,102]. Wang et al. [103] conducted a systematic review of 139 interventions and found evidence of effectiveness (decrease in BMI or BMI *Z*–scores) for school-based interventions with single components—either nutrition or PA—in preventing obesity [103]. On the other hand, Xu et al. [104] found that school-based interventions that incorporated both nutrition education and PA had a positive effect on the reduction of high BP in children. In addition, a statistically significant positive impact on at least one of the seven adiposity-related outcomes in 17 RCTs was found in a study by Bleich et al. [91]. They also found moderate evidence supporting the efficacy of interventions involving multiple settings (e.g., school-based intervention with parental or community involvement) and multiple components (focusing on both nutrition education and physical activity) [91]. Cai et al. [98] conducted a meta-analysis that included 21 intervention studies aimed at children and adolescents that involved a school setting. The results showed an intervention effect of −1.64 mmHg (95% CI: −2.56, −0.71; *p* = 0.001) for SBP and −1.44 mmHg (95% CI: −2.28, −0.60; *p* = 0.001) for DBP. The authors observed that incorporating both diet and PA had significantly greater effects on SBP and DBP than single-component interventions. When comparing the effect among children and adolescents, Dong et al. [105] showed a decrease in SBP, DBP, BMI *Z*–scores, SBP *Z*–scores, and DBP *Z*–scores only in the early age group (up to 12 years of age) as opposed to the older participants. In addition, Brown et al. [92] showed low-certainty evidence of intervention effectiveness with single and multiple components in the age group of 13 to 18, suggesting that early age represents a potential window of opportunity for achieving maximal results. It is important to consider the cumulative impact of multicomponent interventions initiated at an early age, which in turn may contribute to various health aspects in adulthood [102]. However, there is a lack of consensus regarding the recommended duration of interventions [91]. A noteworthy observation is that some studies have reported mixed results, warning that evidence for school-based interventions, their components, and combinations is yet to be determined and fully understood [90].

Community-based interventions

Community-based interventions (CBIs) focused on children and adolescents can influence behavior related to diet and PA, contributing to obesity risk reduction, which is why they have potential [95,106,107]. CBIs with a multicomponent, multisector, multistrategy, and multisetting approach represent a promising model with a moderate effect for childhood obesity prevention linked with cardiometabolic health [103,108]. In addition, a community-based lifestyle intervention implemented during a period of two years showed a decrease of 1.6 mmHg in children as opposed to the control group [104]. In line with this, studies highlighted the importance of community-based interventions that include various layers of society focusing on children such as the school setting, parental involvement, and activities supporting behavioral change [102,108]. However, several studies have shown the limited effect of CBIs on the BMI Z–score [102,109].

Future outlook

Pediatric obesity is a major public health concern that is interlinked with current and future health complications. The prevention of obesity is of utmost importance since obese children have higher SBP and DBP and thus higher risk of CVD in adolescence and adulthood [2]. Studies clearly show that childhood obesity can be prevented through health behavior modifications [98]. There has been abundant research reporting on obesity prevention in children and youth that is focused primarily on diet-related and PA habits [90,92]. School-based interventions, characterized by multiple components, are prevalent [91,105]. Evidence suggests that multicomponent interventions implemented at different levels are associated with decreased body weight and favorable health outcomes related to adiposity [103,108,110]. In addition, research shows that they represent a potent model for improving body weight and cardiometabolic outcomes including BP [98]. In summary, multicomponent and multisetting childhood obesity programs seem to yield positive effects on BP [98]; however, there is a need for a more rigorous evaluation of studies. In addition, studies suggest exploring the effectiveness of intervention components and their combinations [110], incorporating outcomes such as direct adiposity measurements [102], and optimizing intervention effects to advance our understanding and effectiveness of public health strategies [91,102,103,110].

## 9. Summary

Hypertension, or high blood pressure, in children is a serious health concern that can lead to various complications if not properly managed. As this literature review has shown, there are several effective models of nutrition that can be recommended in the prevention and treatment of hypertension. The DASH diet remains the most proven effective strategy for managing HTN. In addition, it is easy to follow and should continue to be the first recommended approach in the fight against hypertension, including in children and adolescents. Other dietary patterns discussed may benefit blood pressure, although information is limited and based mainly on low-quality research. In addition to limiting sodium intake, which is commonly discussed, the role of the appropriate proportions of sodium and potassium intake in diet should be emphasized. One important dietary factor in the development of HTN is also fluid intake. Water should be the first recommended liquid for children, and its adequate amount consumed daily prevents dehydration, thus affecting normal blood pressure. Energy drinks, due to their proven effect on increasing blood pressure, especially when combined with factors like heavy physical exertion or alcohol, pose a serious threat to health and life and should be banned from consumption by children. On the other hand, although increasingly popular among athletes, these drinks are not intended for the nutrition of healthy children. Despite the lack of a direct link to increased blood pressure, they are an additional source of simple sugars, and excessive consumption can lead to obesity. Furthermore, antioxidants in food are available at low concentration, and their bioavailability from eating plant-based-food is low; eating plant-based food cannot lead to an excess of antioxidants, but it can contribute to maintaining a good total antioxidant plasma capacity to cope with physiological free radicals. Furthermore, we have to distinguish between antioxidants from plant-based food and antioxidants obtained by supplementation. Antioxidant supplementation should be considered only in the case of hypovitaminosis and in subjects that cannot eat food containing natural bioactive molecules to avoid any pro-oxidant impact. We critically highlight the negative impact of high level of antioxidants or their inadequate use, specifying the unhealthy impact of inappropriate antioxidant supplementation. In conclusion, diet plays a fundamental role in the prevention of HTN in children and adolescents, and it can support the treatment of hypertension in children. A lot of effort must be made at various levels of public health in order to talk about an effective strategy against hypertension in children.

## Figures and Tables

**Table 1:** Recommended adequate intake of sodium and potassium in different age groups during childhood.

Recommended Adequate Intake [mg/day]					
Age	The European Food Safety Authority (EFSA) Panel on Nutrition		Age	The Food and Drug Administration (US)	
	Sodium	Potassium		Sodium	Potassium
7–11 months	200	750			
1–3 years	1300	800	1–3 years	1500	3000
4–6 years	1700	1100	4–8 years	1900	3800
7–10 years	2000	1800			
11–14 years	2000	2700	9–13 years	2200	4500
15–17 years	2000	3500	14–18 years	2300	4700
